# Unveiling the Anomalous Hall Response of the Magnetic Structure Changes in the Epitaxial MnBi_2_Te_4_ Films

**DOI:** 10.1021/acs.nanolett.3c04095

**Published:** 2024-02-10

**Authors:** Kejing Zhu, Yang Cheng, Menghan Liao, Su Kong Chong, Ding Zhang, Ke He, Kang L. Wang, Kai Chang, Peng Deng

**Affiliations:** † Beijing Academy of Quantum Information Sciences, Beijing 100193, China; ‡ Department of Electrical and Computer Engineering, University of California, Los Angeles, California 90095, United States; § Department of Physics, Tsinghua University, Beijing 100084, China

**Keywords:** topological materials, anomalous Hall effect, antiferromagnetism, molecular beam epitaxy

## Abstract

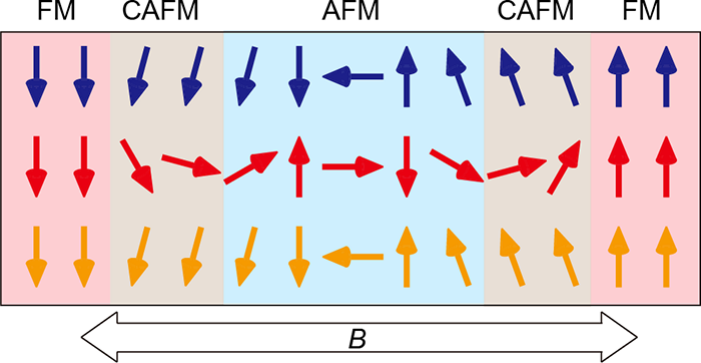

Recently discovered as an intrinsic antiferromagnetic topological insulator, MnBi_2_Te_4_ has attracted tremendous research interest, as it provides an ideal platform to explore the interplay between topological and magnetic orders. MnBi_2_Te_4_ displays distinct exotic topological phases that are inextricably linked to the different magnetic structures of the material. In this study, we conducted electrical transport measurements and systematically investigated the anomalous Hall response of epitaxial MnBi_2_Te_4_ films when subjected to an external magnetic field sweep, revealing the different magnetic structures stemming from the interplay of applied fields and the material’s intrinsic antiferromagnetic (AFM) ordering. Our results demonstrate that the nonsquare anomalous Hall loop is a consequence of the distinct reversal processes within individual septuple layers. These findings shed light on the intricate magnetic structures in MnBi_2_Te_4_ and related materials, offering insights into understanding their transport properties and facilitating the implementation of AFM topological electronics.

MnBi_2_Te_4_ has recently drawn diverse interest in the field of condensed matter physics as an intrinsic antiferromagnetic (AFM) topological insulator.^1−6^ Owning to its intriguing topological and magnetic properties, MnBi_2_Te_4_ exhibits various exotic states, such as quantum anomalous Hall insulators,^7^ axion insulators,^8^ Weyl semimetals,^2,3,9^ etc., which are not only appealing in fundamental research but also have tremendous potential in realistic applications.

The tetradymite MnBi_2_Te_4_ is a van der Waals material with septuple layers (SLs) stacked along the *c*-axis.^10^ The magnetic moment contributed by Mn atoms is A-type AFM ordered in the ground state, i.e., ferromagnetically ordered within each SL, while the neighboring SLs are antiferromagnetically coupled.^5,11^ The magnetic structure varies when subjected to an external magnetic field and influences the properties of AFM topological insulators in a profound manner. The arrangement of the magnetic order determines the symmetry of the system, thereby dictating the topology of the electronic structure. This, in turn, shapes the transport properties of the AFM topological insulators. As such, various exotic phases are realized in MnBi_2_Te_4_ under different magnetic structures. The quantum anomalous Hall effect is realized in MnBi_2_Te_4_ with an odd number of layers that hosts uncompensated surface magnetization.^7^ In contrast, the axion insulator phase is observed in even-layer MnBi_2_Te_4_, in which the magnetization of top and bottom surfaces lies in opposite directions.^8^ Moreover, when subjected to an applied magnetic field, few-layer MnBi_2_Te_4_ with ferromagnetic or canted AFM order gives rise to Chern insulator states with different Chern numbers.^7,8,12−15^ In its bulk form, ferromagnetic MnBi_2_Te_4_ with an out-of-plane magnetic order is predicted to be a type-II Weyl semimetal,^2^ while it transitions to a type-I Weyl semimetal when magnetization direction is pointing in-plane.^9^ Additionally, MnBi_2_Te_4_ with a canted AFM order is proposed to host Mobius insulator and higher-order topological insulator phases.^16^

In transport measurements, the anomalous Hall effect is an effective probe for uncovering the magnetic structure of the material.^17^ The epitaxially grown MnBi_2_Te_4_ films in different reports^4,18−21^ exhibit certain common features in transport: the anomalous Hall resistance saturates at high magnetic fields as the system enters a ferromagnetic state, and at small fields, a nonsquare hysteresis loop is often observed. The nonsquare loop has been attributed to the coexistence of two different anomalous Hall components, which are contributed from the MnBi_2_Te_4_ phase and a secondary phase, respectively, although the type of the secondary phase may differ in different reports.^18,20^ Notably, the magnitude of the anomalous Hall resistance contributed from the secondary phase is comparable to that from MnBi_2_Te_4_. On the other hand, it has been demonstrated via multiple characterization methods that the epitaxially grown films show a predominance of the MnBi_2_Te_4_ phase,^22−24^ the anomalous Hall contributed from the secondary phase should be small, if not absent at all.

In this work, we investigate the anomalous Hall response of the MnBi_2_Te_4_ films upon subjecting them to an external field sweep. The anomalous Hall curves obtained from the 1 SL MnBi_2_Te_4_ displayed a square hysteresis loop, indicative of a single flip process in the magnetization reversal. In the 3 SL MnBi_2_Te_4_, however, a nonsquare hysteresis loop in observed. The nonsquare behavior arises as a consequence of AFM coupling between the SLs. By examining the anomalous Hall resistance, we uncover the magnetic structures of MnBi_2_Te_4_ during the magnetization reversal process, which was further corroborated by our micromagnetic simulations.

The MnBi_2_Te_4_ films are grown using molecular beam epitaxy (MBE),^4,22^ and the details are described in Materials and Methods. The crystal structure of MnBi_2_Te_4_ is schematically shown in Figure 1a, where each SL layer consists of atomic layers stacked in the order Te–Bi–Te–Mn–Te–Bi–Te along the *c*-axis. The epitaxial growth process was monitored using a reflection high electron energy diffraction (RHEED). The RHEED image of 5 SL MnBi_2_Te_4_ is presented in Figure 1b, revealing sharp and streaky patterns indicative of high-quality films. The high quality of the film was further confirmed by X-ray diffraction (XRD) measurement, as shown in Figure 1c. The XRD results exhibit clear peaks corresponding to MnBi_2_Te_4_, while the peaks associated with other secondary phases, such as the most commonly observed Bi_2_Te_3_ phase, are absent, demonstrating the high purity of the sample.

**Figure 1:**
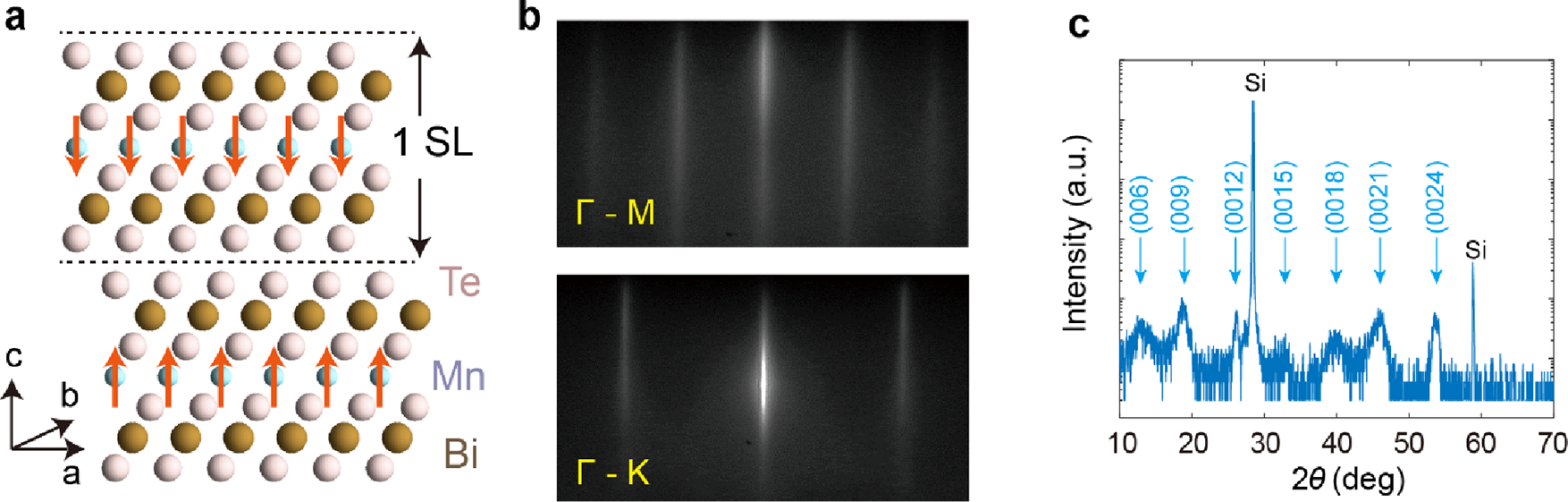
Growth and characterizations of MnBi_2_Te_4_. **a**, Schematics of the atomic structure of MnBi_2_Te_4_. **b**, RHEED images of a MnBi_2_Te_4_ sample along high symmetry azimuths. **c**, XRD result of a 7 SL MnBi_2_Te_4_. The peaks corresponding MnBi_2_Te_4_ to are highlighted by blue arrows.

The MBE grown films are patterned into Hall bars for transport measurement, and the schematic drawing of the measurement setup is shown in the inset of Figure 2a. Figure 2 presents the anomalous Hall resistance (*ρ*_AH_) data measured under a perpendicular magnetic field for MnBi_2_Te_4_ samples with thicknesses ranging from 1 SL to 4 SL (the corresponding field dependences of the longitudinal resistance (*ρ*_*xx*_) and Hall resistance (*ρ*_*xy*_) for the same samples can be found in Figures S1 and S2, respectively). The *ρ*_AH_ was obtained by subtracting the linear background of the Hall resistance contributed by the ordinary Hall effect, i.e., *ρ*_*xy*_ = *ρ*_OH_ + *ρ*_AH_ = ρ_0_*H*_*z*_ + *ρ*_a_*M*_*z*_. Figure 2a shows the anomalous Hall curves for 1 SL MnBi_2_Te_4_ at different temperatures. A square hysteresis loop is clearly seen at low temperatures, an indication of the ferromagnetism in the 1 SL sample. The anomalous Hall curves for 2–4 SL MnBi_2_Te_4_, in contrast, exhibit distinctly different hysteresis loops with multiple transitions (Figure 2b–d), the origin of which will be subject to further discussion later.

**Figure 2:**
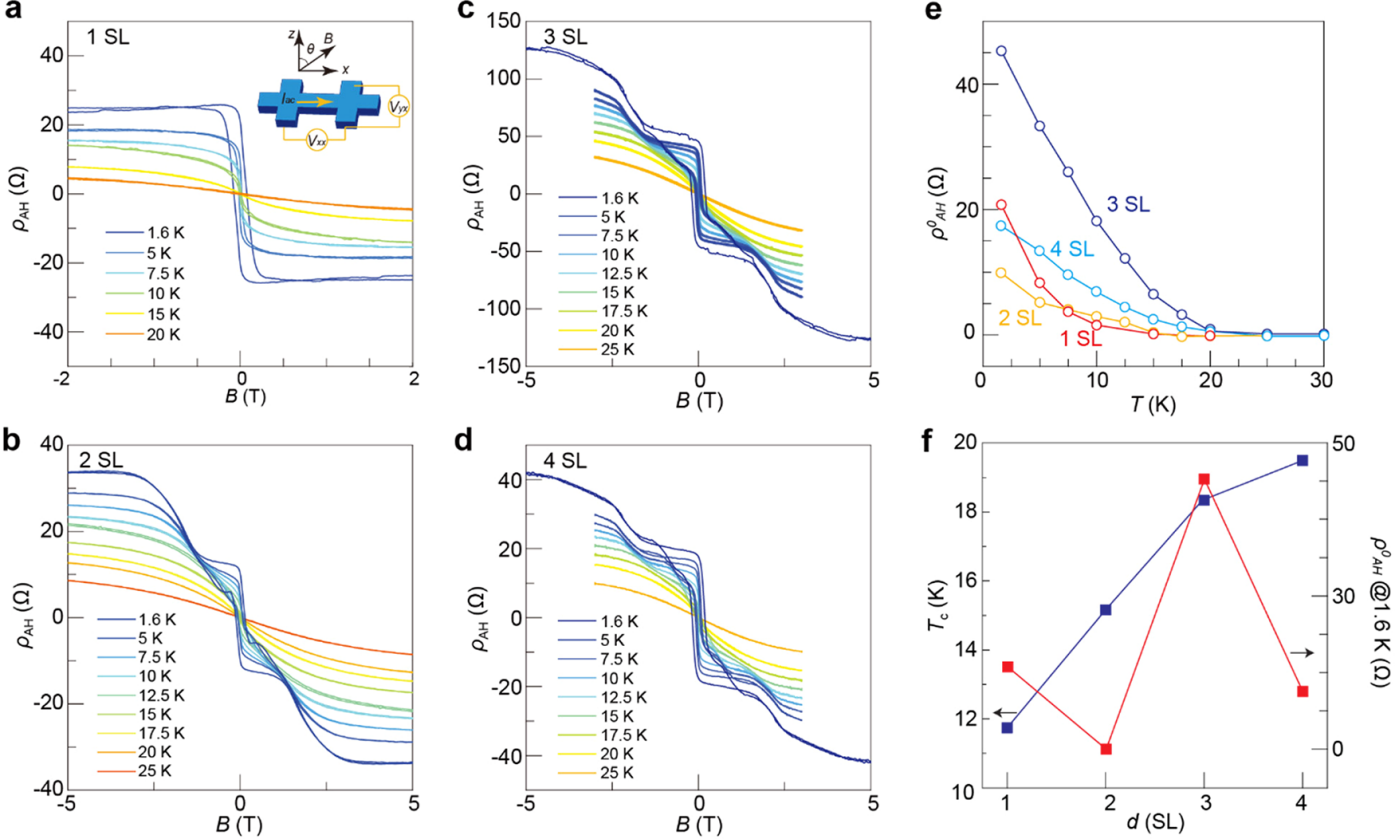
Thickness dependence of the anomalous Hall curves under perpendicular fields. **a**–**d**, Field dependence of *ρ*_AH_ for MnBi_2_Te_4_ films with 1 SL to 4 SL thickness. **e**, Temperature dependence of ρ_AH_^0^ for MnBi_2_Te_4_ films with different thicknesses. **f**, Thickness dependence of *T*_c_ and ρ_AH_^0^ at 1.6 K.

Figure 2e summarizes the thickness dependence of the temperature evolution of the anomalous Hall value at zero magnetic field (ρ_AH_^0^). Upon increasing temperature, ρ_AH_^0^ decreases across all thicknesses. We define *T*_c_ as the temperature at which ρ_AH_^0^ exceeds 5% of its base temperature value. The values of *T*_c_ obtained in this manner are consistent with those acquired from the Arrott plot, as presented in Figure S3. The thickness dependence of *T*_c_ is shown in Figure 2f, where the monotonic rise in *T*_c_ with increasing thickness demonstrates a stronger magnetic coupling in thicker samples. Interestingly, the value of ρ_AH_^0^ at 1.6 K displays oscillatory behavior (Figure 2f). This even–odd oscillation is a direct consequence of the antiferromagnetic ordering inherent to MnBi_2_Te_4_. In an ideal even layer sample, ρ_AH_^0^ would be zero due to the absence of net magnetization. However, achieving a perfect sample with an exact number of 2*n* layers of MnBi_2_Te_4_ is challenging in the MBE growth. The growth, conducted at a relatively high substrate temperature to avoid the formation of Mn-doped Bi_2_Te_3_,^22^ leads to the formation of the 2*n* + 1 layer before the complete coverage of the 2*n* layer. Consequently, odd-layer components unavoidably exist in macroscopic even-layer films, contributing to anomalous Hall signals. Therefore, the value of ρ_AH_^0^ is comparable in the 2 SL and 4 SL samples and is significantly smaller than that in the 3 SL sample, even though the 4 SL sample possesses the highest *T*_c_. For the same reason, the anomalous Hall curves of the 2 and 4 SL MnBi_2_Te_4_ (Figure 2b,d) are qualitatively similar to those in the 3 SL sample (Figure 2c).

Considering that the anomalous Hall resistance in even-layer samples stems from the residual magnetization of the odd-layer component, our following discussion focuses on the anomalous Hall results of the 1 and 3 SL samples and the insights they provide in revealing magnetic structures of MnBi_2_Te_4_. Figure 3a presents the field dependence of *ρ*_AH_ under different θ for the 1 SL MnBi_2_Te_4_, wherein θ is the angle between the directions magnetic field and the sample normal. (The corresponding field dependence of *ρ*_*xy*_ for different θ is shown in Figure S4.) At high magnetic fields, *ρ*_AH_ saturates for all θ. The saturation value of *ρ*_AH_, denoted as ρ_AH_^s^, decreases as θ increases, exhibiting a cosine relationship (Figure 3a inset). That ρ_AH_^s^ is proportional to the out-of-plane component of the magnetic field suggests the direction of the magnetization is fully pinned by the applied field at high fields.

**Figure 3:**
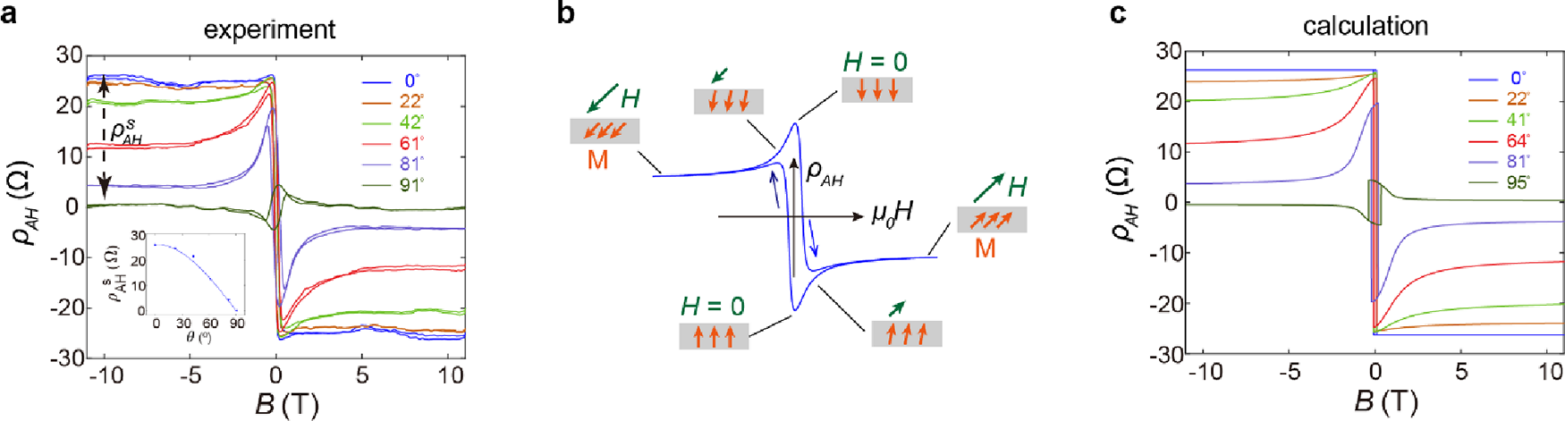
Anomalous Hall curves of 1 SL MnBi_2_Te_4_. **a**, Field dependence of *ρ*_AH_ under different field angles for a 1 SL MnBi_2_Te_4_. All curves were taken at 1.6 K. Inset: angle dependence of ρ_AH_^s^, displaying a cosine relationship. **b**, Schematics of the magnetization direction under a tilted filed. **c**, Calculated results for the field dependence of *ρ*_AH_ under different field angles.

The field dependent *ρ*_AH_ curves in 1 SL of MnBi_2_Te_4_ exhibit distinct hysteresis loops for different field directions. When the applied field is perpendicular to the sample (θ = 0), as discussed above, a square hysteresis loop is observed. The anomalous Hall resistance can be fitted to , where *ρ*_0_ and *H*_c0_ are the amplitude and coercivity of the anomalous Hall loop, respectively, and *H*_0_ is a constant (see fitted results in Figure S6). As the direction of the applied tilts, a hump structure emerges in the hysteresis loop. This behavior can be attributed to the combined effects of the Zeeman energy and perpendicular magnetic anisotropy, as illustrated in Figure 3b. Under the high fields where Zeeman energy dominates, the magnetization lies along the field direction. At zero field, the perpendicular magnetic anisotropy drives the magnetization vertically aligned. Under modest fields, the magnetization tilts in a manner that minimizes the total energy. The observed anomalous Hall response is further corroborated by the model calculation. The total energy for 1 SL MnBi_2_Te_4_ is *E*_total_ = *E*_Zeeman_ + *E*_ani_ = −*MH* cos(α – θ) + *K* sin^2^ θ, wherein *M* is magnetization, *H* is the applied field, *K* is the uniaxial anisotropy energy constant, and θ and α are the tilting angles of *M* and *H*, respectively. The calculation results are presented in Figure 3c, displaying good agreement with the experiment results. Note that in the discussion of anomalous Hall resistance of the 1 SL MnBi_2_Te_4_, no contribution from any secondary phase is involved.

Moving forward, we examine the anomalous Hall behavior in the 3 SL MnBi_2_Te_4_ sample. The field dependence of *ρ*_AH_ at the base temperature for the 3 SL MnBi_2_Te_4_ sample is presented in Figure 4a. In contrast to the 1 SL case, the anomalous Hall curve of 3 SL MnBi_2_Te_4_ exhibits a nonsquare hysteresis loop in which multiple steps are observed between two plateaus under saturated magnetization. That the nonsquare loop appears only in the 3 SL (as well as in 2 SL and 4 SL) sample but not the 1 SL one suggests it is not due to the existence of certain secondary phases in the material; rather, it indicates a more complicated magnetization reversal process in the multilayer MnBi_2_Te_4_. This complexity arises due to the AFM coupling between the SLs, which competes with ferromagnetic alignment forced by external magnetic field. Specifically, at high magnetic fields, the magnetization in all three SLs aligns along the applied field, while at zero magnetic field, the magnetization in the middle layer points in the opposite direction compared to the top and bottom layers, as schematically illustrated in Figure 4a. Therefore, during a full magnetization reversal process, the magnetization in the top and bottom layers flips once, while the magnetization in the middle layer undergoes three flips.

**Figure 4:**
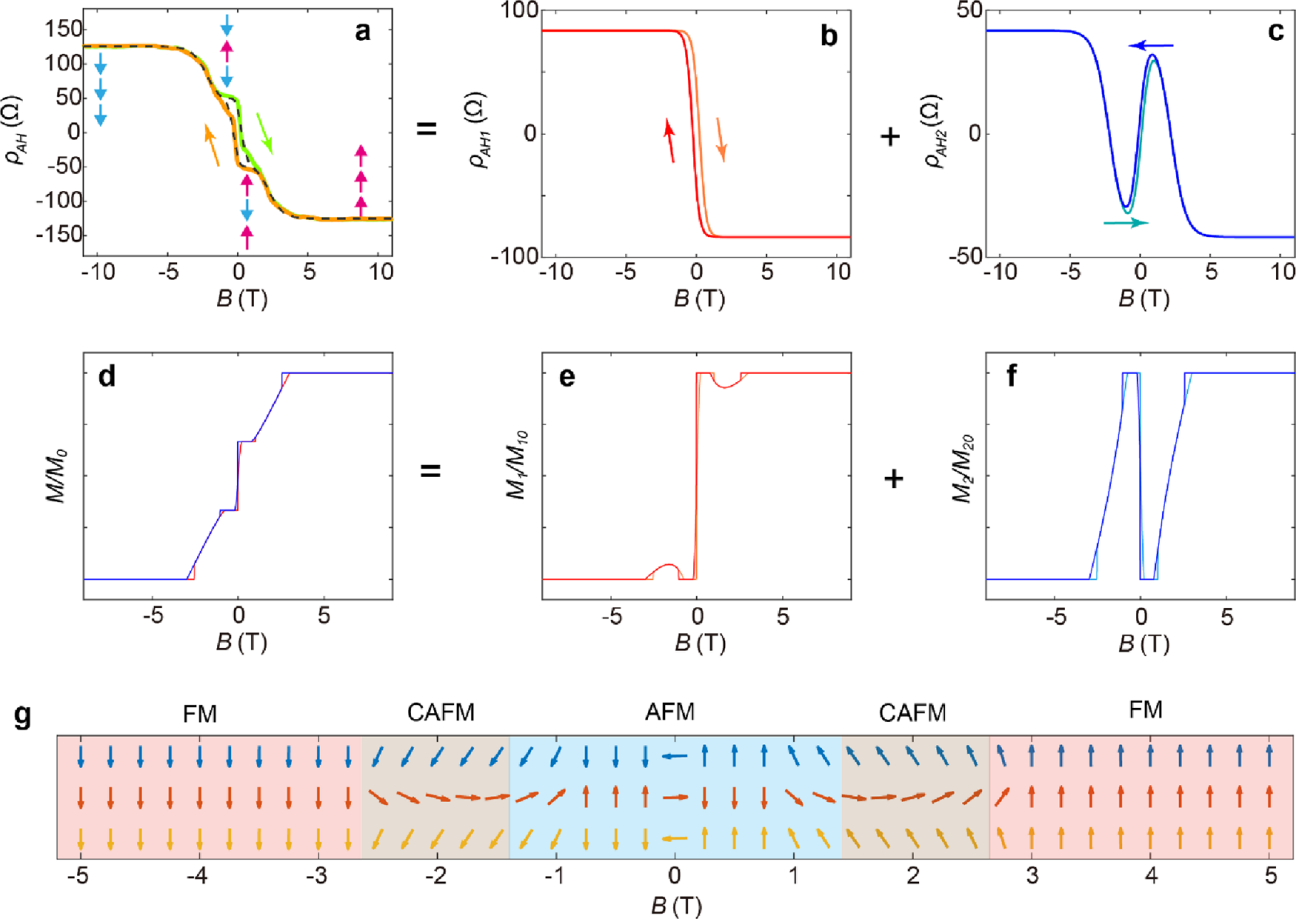
Anomalous Hall curves of 3 SL MnBi_2_Te_4_ under a perpendicular field. **a**, Field dependence of *ρ*_AH_ for a 3 SL MnBi_2_Te_4_ taken at 1.6 K. The field is applied perpendicular to the sample surface. The dashed line is the fitted results. *ρ*_AH_ can be decomposed into two components contributed by **b**, the top and bottom layers, and **c**, the middle layers. These layers undergo different reversal process, as schematically shown by the arrows in **a**. **d**, Microsimulation results of field dependence of magnetization for a 3 SL MnBi_2_Te_4_. **e**, Simulated field dependence of magnetization for the top/bottom layer. **f**, Simulated field dependence of magnetization for the middle layer. **g**, Simulated magnetic structure during the reversal process.

The anomalous Hall curve therefore can be decomposed into two components: *ρ*_AH_ = ρ_AH1_ + ρ_AH2_; here ρ_AH1_ represents the contribution from the top and bottom layers and ρ_AH2_ is the contribution from the middle layer. The two components can be fitted with the expressions ρ_AH1_ =  and ρ_AH2_ = , respectively, to showcase the one-time and three-time flips of the reversal process in the corresponding layers. Here, ρ_0_ is the amplitude of anomalous Hall contributed by a single SL, *H*_c1_ is the coercivity for the top/bottom layer, *H*_c21_, *H*_c22_, and *H*_c23_ are the field values when the magnetization of the middle layer undergoes a flip, and finally, *H*_1_, *H*_21_, *H*_22_, and *H*_23_ are constants. The two components are displayed in Figure 4b,c, respectively. The fitted result of *ρ*_AH_ is highlighted by dashed lines in Figure 4a.

To gain further insights into the intricate magnetic structure during the magnetization reversal process, we conducted micromagnetic simulations of 3 SL MnBi_2_Te_4_. The simulated result of the out-of-plane component of the magnetization is presented in Figure 4d, showing good agreement with the anomalous Hall resistance obtained from the transport measurements. Furthermore, we examine the out-of-plane component for each individual SL during the field sweep process. The contributions from the top/bottom and middle layers, denoted as *M*_1_ and *M*_2_, respectively, are shown in Figure 4e,f. As expected, the magnetization in the top and bottom layers undergoes a single flip, while the magnetization in the middle layer experiences three successive flips throughout the entire reversal process. Notably, *M*_1_ deviates from its saturation value in the field range of approximately 1 T < |*B*| < 3 T, indicating the canted magnetization in the top and bottom layers. This intriguing behavior is mirrored by the occurrence of the first flip in the middle layer within the same field range. The deviation can be attributed to the spin-flop transition as the magnetization transitions between the canted AFM state and AFM state.^13,18,25^ In Figure 4g, we present a schematic drawing of the magnetic structure for the entire reversal process.

Figure 5 presents the field dependence of *ρ*_AH_ for 3 SL MnBi_2_Te_4_ under different rotation angles. As the field direction changes from out-of-plane to in-plane, systematical changes are revealed in the anomalous Hall curves. Upon increasing θ, ρ_AH_^s^ decreases monotonically, displaying a cosine dependence (Figure 5f). This indicates that in 3 SL MnBi_2_Te_4,_ the direction of magnetization is fully pinned by the applied field at high fields. As the field decreases to zero, the magnetization becomes AFM ordered along the vertical direction. As such, the anomalous Hall at zero field, ρ_AH_^0^, maintains almost the same value as θ varies between 0° and 80°. Interestingly, when θ = 90°, the value of ρ_AH_^0^ is significantly reduced. This suggests that when pinned by an in-plane field, the magnetization does not fully restore the out-of-plane AFM alignment when the field is removed. As long as θ ≠ 90°, the out-of-plane component of the field breaks the symmetry and will force the magnetization to align along the vertical direction when approaching the zero field, resulting in a noticeable increase in ρ_AH_^0^.

**Figure 5:**
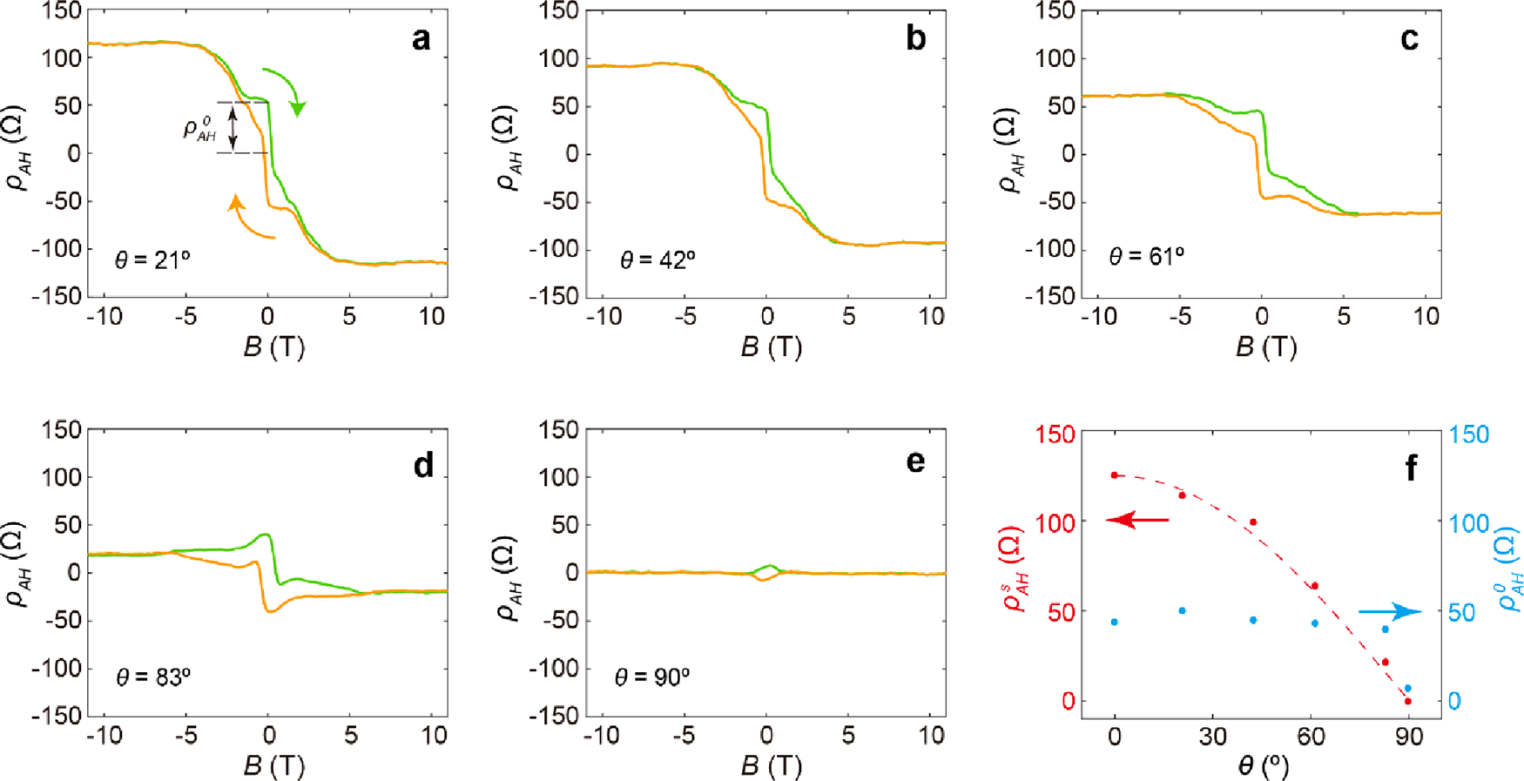
Angle-dependent anomalous Hall curves in the 3 SL MnBi_2_Te_4_. **a**–**e**, Field dependence of *ρ*_AH_ for a 3 SL MnBi_2_Te_4_ under different field angles. All data were taken at 1.6 K. **f**, Field angle dependent anomalous Hall resistance at saturation field, ρ_AH_^s^, and zero field, ρ_AH_^0^, respectively.

As discussed above, the nonsquare hysteresis loop in the anomalous Hall curve reveals the complex evolution of the magnetic structure in the MnBi_2_Te_4_, which is subject to both the applied field and its intrinsic AFM ordering. Notably, the contribution from any secondary phase is not included in the discussion. Such a finding not only provides insights into the understanding of the magnetic structure of MnBi_2_Te_4_ but also may explain similar transport behaviors observed in broader material systems that are closely related to MnBi_2_Te_4_, such as the heterostructure of MnTe/Bi_2_Te_3_ and Mn-doped Bi_2_Te_3_.^26−28^ Given that MnBi_2_Te_4_ naturally forms during the epitaxial growth of MnTe/Bi_2_Te_3_ heterostructures or Mn-doped Bi_2_Te_3_, it may be responsible for the nonsquare loops in the anomalous Hall effect of these materials as well.

In conclusion, we carried out transport measurement on epitaxial MnBi_2_Te_4_ films with different thicknesses, and the anomalous Hall results reveal the intricate magnetization structure that arises due to the interplay of the applied field and its intrinsic AFM ordering. The observed nonsquare behavior in the anomalous Hall curves indicates the presence of multiple contributions from different SLs with distinct reversal processes. These findings are of significant importance as they provide insight into the intricate magnetic structures of MnBi_2_Te_4_, the exploration of which is crucial for understanding the exotic phases and unique properties exhibited by this material. Our work paves the way for the development of novel technologies based on AFM topological insulators.

## Materials and Methods

### Sample Growth and Characterizations

The growth of the MnBi_2_Te_4_ films was conducted in a home-built MBE chamber with a base vacuum rate of 1 × 10^–10^ Torr. The semiinsulating Si (111) wafers were used as the substrate. Prior to the growth, Si(111) was flashed to 1200 °C to remove the oxides. During the growth, the substrate temperature was held at 240 °C, and high purity Mn (99.9999%), Bi (99.9999%), and Te (99.9999%) were evaporated using standard Knudsen cells. After the growth, a few layers of Bi_2_Te_3_ were capped for protection. The growth was *in situ* monitored by an RHEED. The high-resolution XRD was performed using a PANalytical X’Pert Pro X-ray powder diffractometer with Cu Kα radiation (λ = 1.5406 Å).

### Transport Measurements

Transport measurements were conducted in an Oxford TeslatronPT system. A 1 μA AC current was sourced, and the voltage is picked up by standard lock-in amplifiers (SR830). The longitudinal and Hall resistances are symmetrized and antisymmetried, respectively. The rotation angle was calibrated by the ordinary Hall resistance.

### Micromagnetic Simulations

Micromagnetic simulations were performed using the standard OOMMF^29^ extensible solver (OXS). To achieve the construction of A-type AFM MnBi_2_Te_4_, we set the in-plane ferromagnetic intralayer coupling with positive exchange stiffness *A*_in_ and the out-of-plane antiferromagnetic interlayer coupling with negative exchange stiffness *A*_out_. The system size in simulation is 100 × 100 × 3 nm^3^ for a total of 3 layers with mesh size 5 × 5 × 1 nm^3^. The material parameters are set with intralayer exchange stiffness *A*_in_ = 5 × 10^–14^ J/m, interlayer exchange stiffness *A*_out_ = −1 × 10^–13^, the saturation magnetization (*M*_s_ = 1.4 × 10^5^ A/m) and the perpendicular anisotropy Ku = 2 × 10^4^ J/m^3^.

## Supporting Information Available

Transport results for MnBi_2_Te_4_ films of thickness from 1 SL to 4 SL at different temperatures; Arrott plots for MnBi_2_Te_4_ films of thickness from 1 SL to 4 SL; angle dependent results for odd-layer MnBi_2_Te_4_ films; fitted results for the anomalous Hall loop in the 1 SL MnBi_2_Te_4_; angle dependent anomalous Hall results for 2 SL and 4 SL MnBi_2_Te_4_ (PDF)
