# Supplementary material for: Unveiling the Anomalous Hall Response of the Magnetic Structure Changes in the Epitaxial MnBi2Te4 Films
Source: Nano Lett. 2024 Feb 10;24(7):2181–7. doi: 10.1021/acs.nanolett.3c04095 (PMC10885191; doi:10.1021/acs.nanolett.3c04095)
Supplement: Supplementary file 1 — nl3c04095_si_001.pdf [file nl3c04095_si_001.pdf]

Supplementary materials for

Unveiling the anomalous Hall response of the magnetic structure changes in the epitaxial $MnBi_2Te_4$ films

Kejing Zhu[1†], Yang Cheng[2†], Menghan Liao[3†], Su Kong Chong[1], Ding Zhang[1,3], Ke He[1,3], Kang L. Wang[2], Kai Chang[1], and Peng Deng[1*]

[1]*Beijing Academy of Quantum Information Sciences, Beijing, 100193, China*

[2]*Department of Electrical and Computer Engineering, University of California, Los Angeles, California, 90095, United States*

[3]*Department of Physics, Tsinghua University, Beijing, 100084, China*

[*]Corresponding author: dengpeng@baqis.ac.cn (P.D.)

Table of contents:

S1. Transport results for $MnBi_2Te_4$ films of thickness from 1 SL to 4 SL at different temperatures.

S2. Arrott plots of $MnBi_2Te_4$ films of thickness from 1 SL to 4 SL.

S3. Angle dependent results for odd-layer $MnBi_2Te_4$ films.

S4. Fitted results for the anomalous Hall loop in the 1 SL $MnBi_2Te_4$.

S5. Angle dependent anomalous Hall results for 2 SL and 4 SL $MnBi_2Te_4$.

S1. Transport results for 1 SL – 4 SL $MnBi_2Te_4$ films at different temperatures

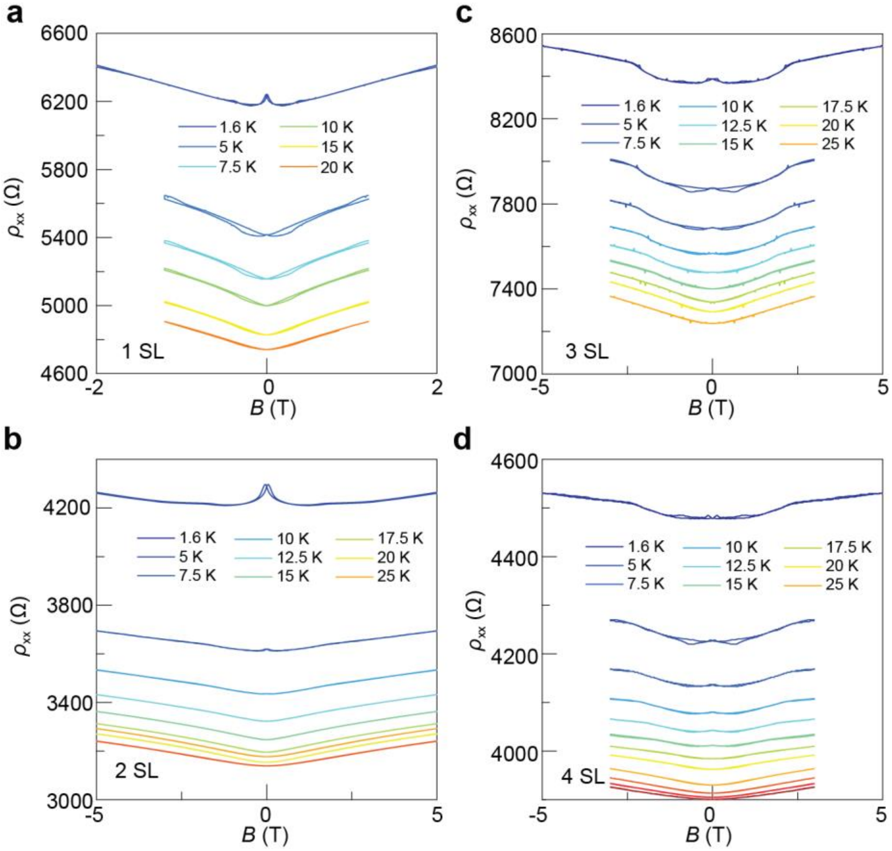

Figure S1. Field dependence of ρ_{xx} at different temperatures for $MnBi_2Te_4$ films of thickness from 1 SL to 4 SL.

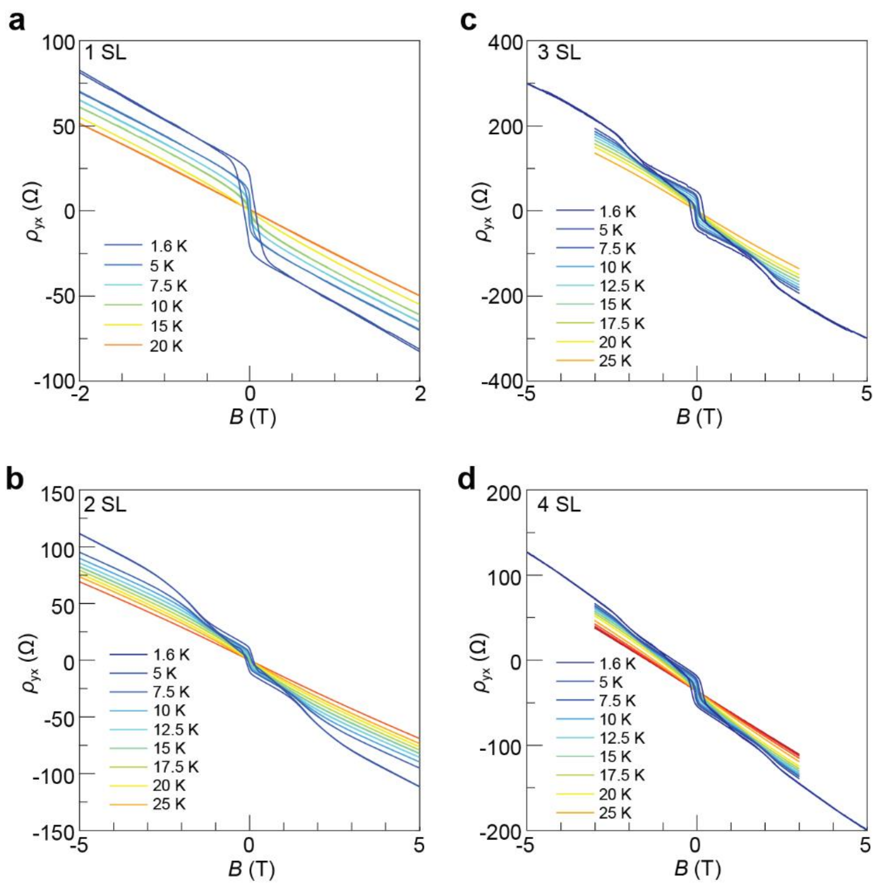

Figure S2. Field dependence of ρ_{xy} at different temperatures for $MnBi_2Te_4$ films of thickness from 1 SL to 4 SL.

S2. Arrott plots for $MnBi_2Te_4$ films of thickness from 1 SL to 4 SL

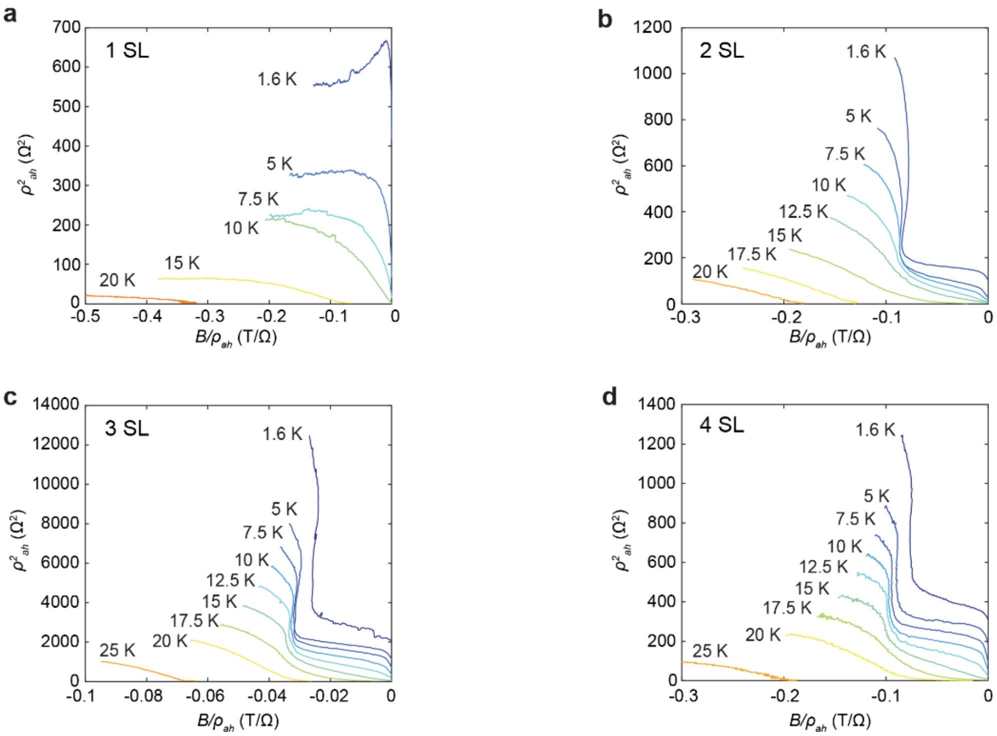

Figure S3. Arrott plot for $MnBi_2Te_4$ films of thickness from 1 SL to 4SL.

S3. Angle dependent results for 1 SL $MnBi_2Te_4$.

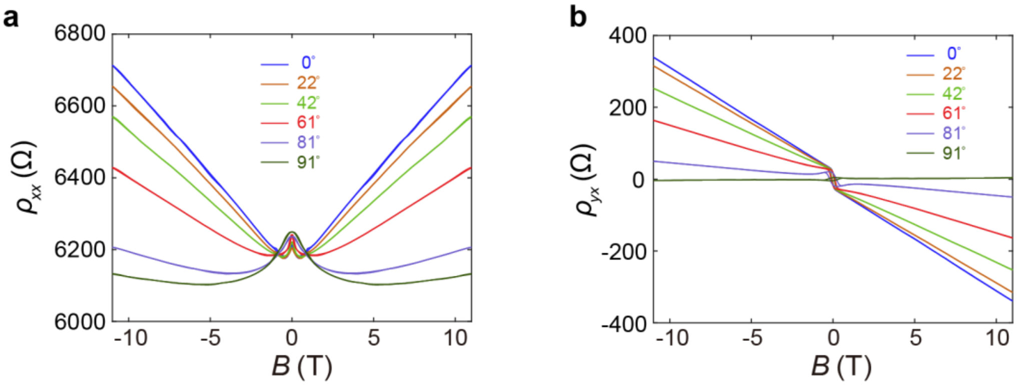

Figure S4. Field dependence of **a**, ρ_{xx} and **b**, ρ_{xy} for 1 SL $MnBi_2Te_4$ under different θ.

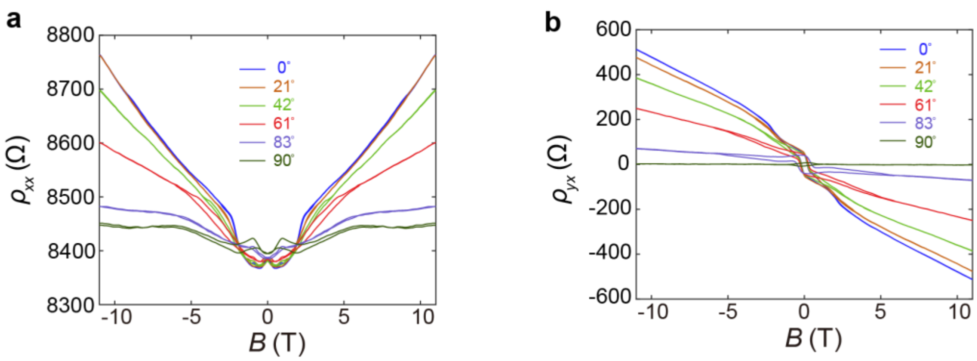

Figure S5. Field dependence of **a**, ρ_{xx} and **b**, ρ_{xy} for for the 3 SL $MnBi_2Te_4$ under different θ.

S4. Square anomalous loop in the 1 SL $MnBi_2Te_4$.

Figure S6 presents the field dependence of the anomalous loop for the 1 SL under a perpendicular field. The square anomalous Hall loops can be fitted by a hyperbolic tangent function $\rho_{AH} = \rho_0 \tanh(\frac{H-H_{c0}}{H_0})$. This suggests the contribution to the anomalous Hall from other secondary phases is neglectable.

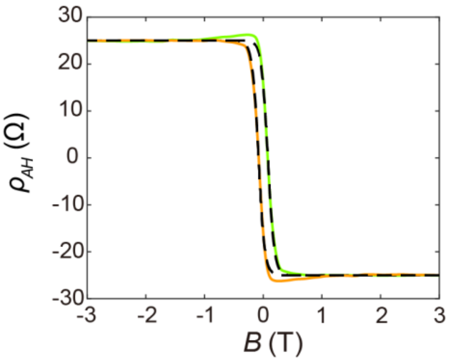

Figure S6. Field dependence of ρ_{AH} for a 1 SL $MnBi_2Te_4$ under a perpendicular field. The curves are taken at 1.6 K. The fitted results are highlighted by dashed lines.

S5. Angle dependent anomalous Hall results for 2 SL and 4 SL $MnBi_2Te_4$.

As discussed in the main text, due to this imperfection in the growth, and since the even layer sample itself does not contribute to the anomalous Hall effect, the anomalous Hall resistance in even layer samples is contributed by the odd layer components. Figures S7 and S8 present the field dependent results of the anomalous Hall resistance for 2 SL and 4 SL $MnBi_2Te_4$, respectively. As can be seen, ρ_{AH}^{S} in 2 SL and 4 SL samples are comparable, and are significantly smaller than that in 3 SL $MnBi_2Te_4$.

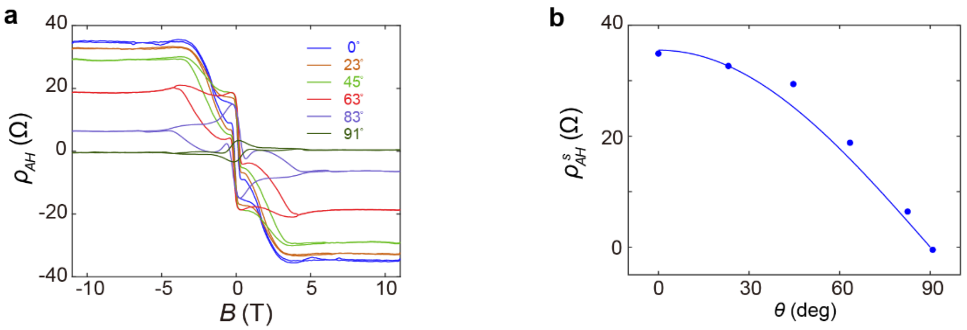

Figure S7. **a**, Field dependence of ρ_{AH} for the 2 SL $MnBi_2Te_4$ under different θ. **b**, Field angle dependent anomalous Hall resistance at saturation field.

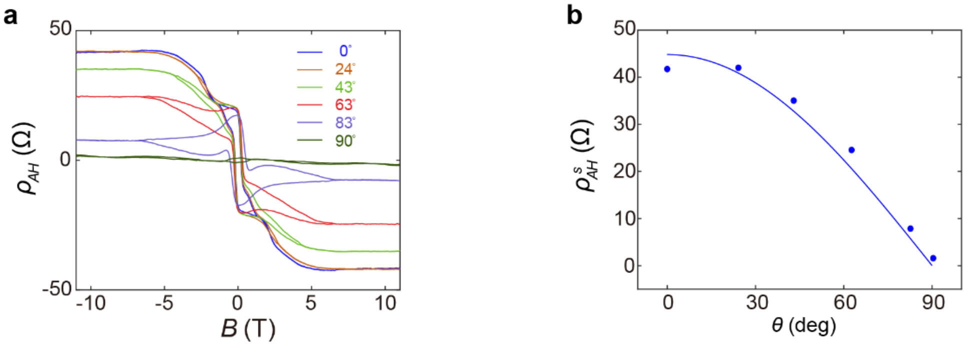

Figure S8. **a**, Field dependence of ρ_{AH} for the 4 SL $MnBi_2Te_4$ under different θ. **b**, Field angle dependent anomalous Hall resistance at saturation field.
